# Supplementary material for: An Emerin LEM-Domain Mutation Impairs Cell Response to Mechanical Stress
Source: Cells. 2019 Jun 10;8(6):570. doi: 10.3390/cells8060570 (PMC6628311; doi:10.3390/cells8060570)
Supplement: Supplementary file 1 [file cells-08-00570-s001.pdf]

**Resolution range**	40.74 - 2.26 (2.341 - 2.26)
**Space group**	C 1 2 1
**Unit cell**	80.2 81.49 64.88 90 121.37 90
**Total reflections**	73970 (6634)
**Unique reflections**	16448 (1524)
**Multiplicity**	4.5 (4.4)
**Completeness (%)**	98.04 (91.75)
**Mean I/sigma(I)**	11.74 (1.77)
**Wilson B-factor**	43.77
**R-merge**	0.09554 (0.7352)
**R-meas**	0.1081 (0.8347)
**R-pim**	0.04968 (0.3885)
**CC1/2**	0.997 (0.658)
**CC***	0.999 (0.891)
**Reflections used in refinement**	16441 (1523)
**Reflections used for R-free**	822 (76)
**R-work**	0.1908 (0.2857)
**R-free**	0.2258 (0.3070)
**CC(work)**	0.956 (0.728)
**CC(free)**	0.942 (0.637)
**Number of non-hydrogen atoms**	2754
**macromolecules**	2651
**ligands**	20
**solvent**	83
**Protein residues**	335
**RMS(bonds)**	0.014
**RMS(angles)**	1.66
**Ramachandran favored (%)**	97.86
**Ramachandran allowed (%)**	2.14
**Ramachandran outliers (%)**	0.00
**Rotamer outliers (%)**	5.86
**Clashscore**	2.27
**Average B-factor**	47.87
**macromolecules**	47.09
**ligands**	162.61
**solvent**	44.88

Suppl. Table 1. Data collection and refinement statistics.
Statistics for the highest-resolution shell are shown in parentheses.
